# Successful management of visceral disseminated varicella zoster virus infection during treatment of membranous nephropathy: a case report

**DOI:** 10.1186/s12879-019-4193-y

**Published:** 2019-07-15

**Authors:** Yoshitaka Furuto, Mariko Kawamura, Akio Namikawa, Hiroko Takahashi, Yuko Shibuya

**Affiliations:** Department of Hypertension and Nephrology, NTT Medical Centre Tokyo, 5-9-22, Higasi-Gotanda, Shinagawa-ku, Tokyo, 141-8625 Japan

**Keywords:** Varicella zoster virus, Visceral disseminated varicella zoster virus infection, Nephrotic syndrome, Membranous nephropathy, Immunosuppressive therapy

## Abstract

**Background:**

Visceral disseminated varicella zoster virus (VDVZV) infection is a rare disease with a high mortality rate (55%) in immunocompromised patients, but it is not yet widely recognized in the field of nephrology. We report a case of VDVZV contracted during immunosuppressive therapy for membranous nephropathy.

**Case presentation:**

A 36-year-old woman was diagnosed with membranous nephropathy and was being treated with immunosuppressive therapy consisting of 60 mg/day prednisolone, 150 mg/day mizoribine, and 150 mg/day cyclosporine. Nephrosis eased; therefore, the prednisolone dosage was reduced. However, 50 days after starting immunosuppressive therapy, the patient suddenly developed strong and spontaneous abdominal pain, predominantly in the epigastric area, without muscular guarding or rebound tenderness. Blood data indicated neutrophil-dominant elevated white blood cell count, reduced platelet count, elevated transaminase and lactate dehydrogenase, slightly increased C-reactive protein, and enhanced coagulability. Abdominal computed tomography revealed a mildly increased enhancement around the root of the superior mesenteric artery with no perforation, intestinal obstruction, or thrombosis. The cause of the abdominal pain was unknown, so the patient was carefully monitored and antibiotic agents and opioid analgesics administered. The following day, blisters appeared on the patient’s skin, which were diagnosed as varicella. There was a marked increase in the blood concentration of VZV-DNA; therefore, the cause of the abdominal pain was diagnosed as VDVZV. Treatment with acyclovir and immunoglobulin was immediately started, and the immunosuppressive therapy dose reduced. The abdominal pain resolved rapidly, and the patient was discharged 1 week after symptom onset.

**Discussions and conclusions:**

This patient was VZV-IgG positive, but developed VDVZV due to reinfection. Abdominal pain due to VDVZV precedes the skin rash, which makes it difficult to diagnose before the appearance of the rash, but measuring the VZV-DNA concentration in the blood may be effective. Saving the patient’s life requires urgent administration of sufficient doses of acyclovir and reduced immunosuppressive therapy.

## Background

Varicella zoster virus (VZV) infection is often experienced by patients who are in an immunocompromised state. The incidence of herpes zoster in chronic kidney disease patients is 12.4/1000 people, several times higher than in healthy individuals [1]. Visceral disseminated VZV (VDVZV) infection is a rare disease with a high mortality rate that occurs in immunocompromised patients undergoing immunosuppressive therapy for blood diseases, kidney transplant recipients, patients with uncontrolled diabetes, and patients with collagen and/or kidney diseases. The symptoms are intense, beginning with sudden onset abdominal pain, and the condition tends to be serious. Early diagnosis and treatment are crucial for patient survival [2–9]. This disease is well known in the field of hematological diseases, but there is still little awareness in the field of nephrology. Recent expansion of various types of immunosuppressive therapy necessitates awareness of VDVZV infection and implementation of appropriate measures to prevent death from this disease in immunocompromised patients. Here we report the case of a patient who developed VDVZV infection approximately 50 days after starting immunosuppressive therapy (consisting of prednisolone, cyclosporine, and mizoribine) for membranous nephropathy. Her condition rapidly became serious, but with appropriate diagnosis and management we were able to save the patient.

## Case presentation

### Case: a 36-year-old woman

#### Primary complaint: intense epigastric pain

History of current condition: The patient became aware of general malaise in August, 2011. She was seen at the Department of Hypertension and Nephrology, NTT Medical Centre, Tokyo in September, and was hospitalized with nephrotic syndrome. A kidney biopsy resulted in the diagnosis of idiopathic membranous nephropathy (Stage II). Serological tests for hepatitis B and C virus were negative. Treatment with 50 mg/day prednisolone was initiated on Day 14 of hospitalization, but the condition was refractory and so this was increased to 60 mg/day on Day 29. Mizoribine (150 mg/day) was added to the treatment regimen on Day 28, and cyclosporine (150 mg/day) added on Day 49. Nephrosis improved thereafter, and the prednisolone dosage was reduced to 40 mg/day. Abdominal pain developed suddenly on Day 77, which worsened and became intense, requiring treatment with opioid analgesics on Day 79.

#### Medical history, family history, allergy history: nothing of note

Physical findings on Day 79: height 168 cm, weight 65.9 kg, body mass index 23.4 kg/m^2^, blood pressure 124/86 mmHg, heart rate 66 bpm and regular, body temperature 37.0 °C. The patient was conscious and lucid and no nuchal rigidity, palpebral conjunctiva anemia, bulbar conjunctiva jaundice, swelling of the lymph nodes, intraoral findings, enlargement of the thyroid gland, or cardiopulmonary noise were noted. The abdomen was flat and soft with normal peristaltic sounds, strong spontaneous pain and tenderness mainly in the epigastric area, but no muscle guarding or rebound tenderness. The pain score was 10 on the Numerical Rating Scale. No costovertebral angle percussion pain. Bilateral edema was observed, but no joint pain or cutaneous findings were present. The patient was not hypertensive.

**Fig. 1:**
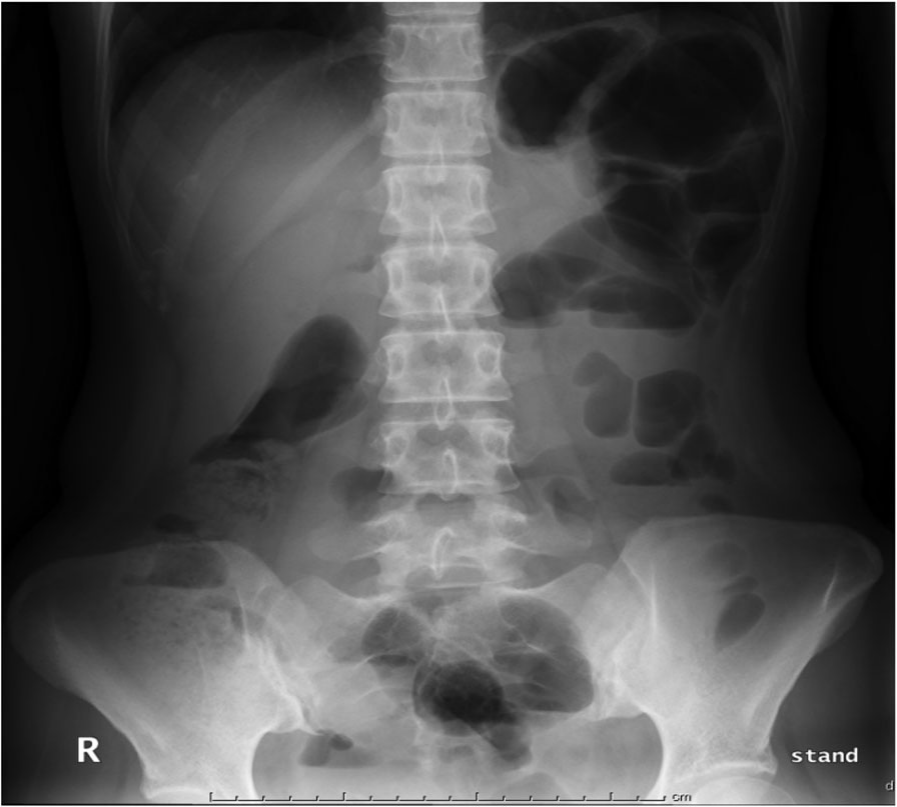
Abdominal x-ray findings: Niveau formation

**Fig. 2:**
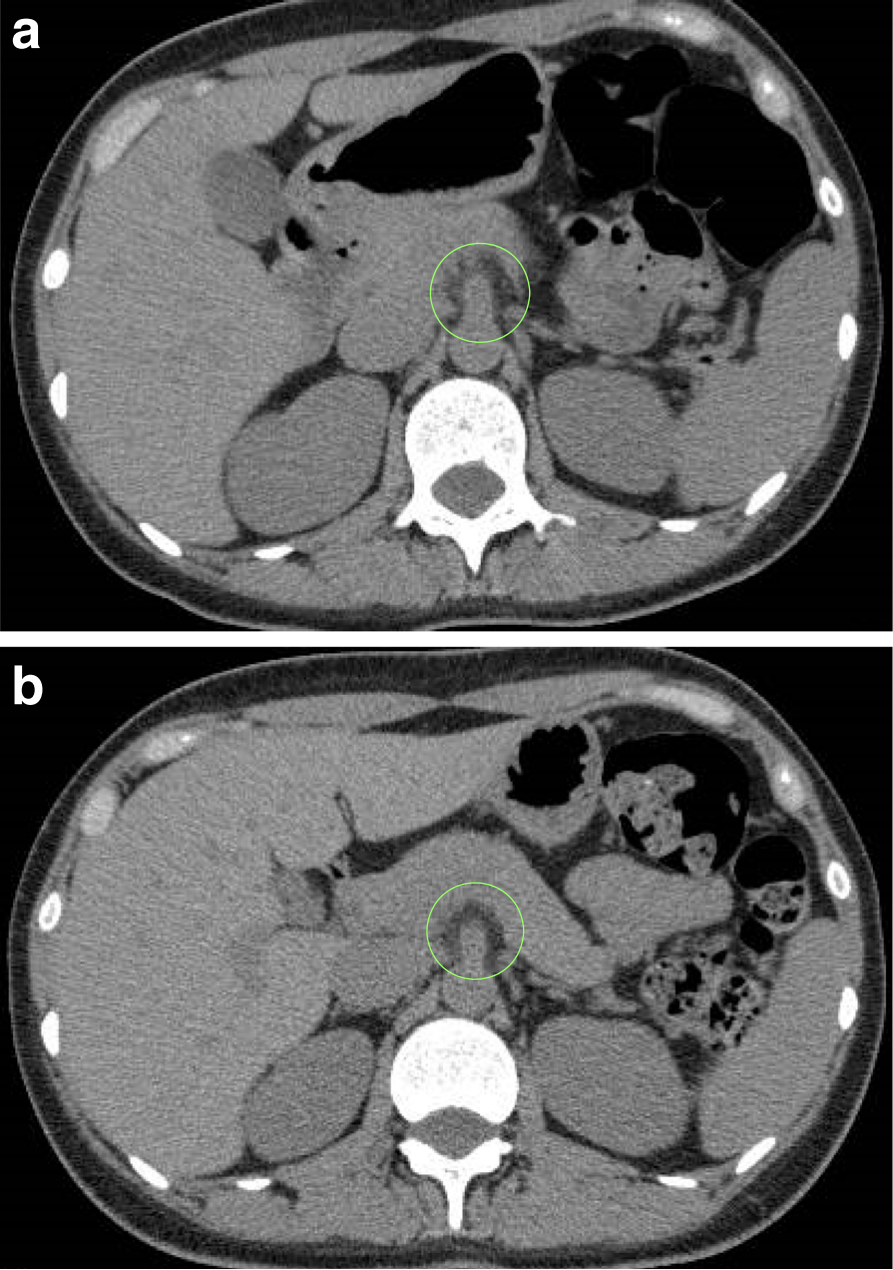
**a** Abdominal computed tomography (CT) findings on Day 79: Mild increases in CT values were seen around the celiac artery and the root of the superior mesenteric artery. **b** Abdominal CT on Day 104: The findings compared with those of Day 79 have changed to approach normal levels

Laboratory tests on Day 79 revealed a neutrophil-dominant elevated white blood cell count, a slightly reduced platelet count, reduced total protein/albumin levels, elevated transaminase and lactate dehydrogenase levels, slightly increased C-reactive protein levels, hypogammaglobulinemia, and enhanced coagulability (Table 1). There was no apparent hypocomplementemia or double stranded DNA antibodies, and tests for anti-proteinase-3-anti-neutrophil cytoplasmic antibody and anti-myeloperoxidase-anti-neutrophil cytoplasmic antibodies were all negative. Urinary protein was 420 mg/day and negative for occult blood. There were no electrocardiogram abnormalities and no notable abnormalities in the chest x-ray images, but niveau formation was observed on the abdominal x-ray (Fig. 1). Abdominal computed tomography (CT) (Fig. 2a) revealed a mildly increased fat tissue density around the celiac artery and root of the superior mesenteric artery, but there were no other abnormalities of note and no findings suggestive of perforation, intestinal obstruction, or thrombosis.

**Table 1:** Laboratory data

Urinalysis/Blood test		Biochemistry/Immunological test/Coagulation test			
Protein	±	TP	4.7 g/dL	HbA1c	5.0%
Occult blood	±	Alb	2.2 g/dL	TSH	1.344 μIU/mL
Red blood cell	1–4/HPF	UA	7.1 mg/dL	FT4	0.85 ng/dL
Protein content	0.42 g/gCr	BUN	8.6 mg/dL	IgG	452 mg/dL
Complete blood cell count		Cr	0.66 mg/dL	IgA	348 mg/dL
White blood cell	10,600/μL	eGFR	81 mL/min/1.73 m^2^	IgM	118 mg/dL
Neutrophil	9080 (85.8%)	TB	0.4 mg/dL	C3	89 mg/dL
Lymphocyte	1190 (11.2%)	AST	105 IU/L	C4	15.1 mg/dL
Monocyte	2.6%	ALT	92 IU/L	CH50	32 U/mL
Eosinophil	0%	ALP	142 IU/L	Antinuclear Ab	1:80
Red blood cell	418 × 10^4^/μL	γ-GT	28 IU/L	Anti-dsDNA-Ab	(−)
Hemoglobin	13.7 g/dL	LDH	581 IU/L	Anti-Sm-Ab	(−)
Hematocrit	39.6%	CK	107 IU/L	Anti-MPO-ANCA	(−)
Platelet	14.8 × 10^4^/μL	Na	139 mEq/L	Anti-PR3-ANCA	(−)
		K	4.0 mEq/L	PT	121%
		Cl	104 mEq/L	PT-INR	0.9
		cCa	9.7 mg/dL	APTT	29.7 s
		IP	3.4 mg/dL	Fibrinogen	242 mg/dl
		CRP	0.4 mg/dL	FDP	18.8 μg/ml
		Glu	96 mg/dl	D-dimer	11.1 μg/ml

*TP* total protein, *Alb* albumin, *UA* uric acid, *BUN* blood urea nitrogen, *Cr* creatinine, *eGFR* estimated glomerular filtration rate, *TB* total bilirubin, *AST* aspartic aminotransferase, *ALT* alanine aminotransferase, *ALP* alkaline phosphatase, *γ-GT* γ-glutamyltransferase, *LDH* lactate dehydrogenase, *CK* creatine kinase, *Na* sodium, *K*: potassium, *Cl* chlorine, *cCa* corrected calcium, *IP* inorganic phosphorus, *CRP* C-reactive protein, *Glu* glucose, *HbA1c* hemoglobin A1c, *TSH* thyroid stimulating hormone, *FT4* free thyroxine, *IgG* immunoglobulin G, *IgA* immunoglobulin A, *IgM* immunoglobulin M, *Ab* antibody, *dsDNA* double-stranded DNA, *Sm* Smith, *MPO-ANCA* myeloperoxidase-anti-neutrophil cytoplasmic antibody, *PR3-ANCA* proteinase-3-anti-neutrophil cytoplasmic antibody, *PT* prothrombin time, *PT-INR* prothrombin time-international normalized ratio, *APTT* activated partial thromboplastin time, *FDP* fibrinogen degradation products

**Fig. 3:**
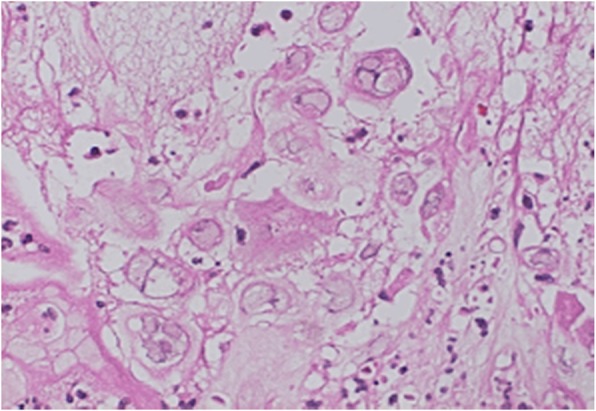
Lesion skin biopsy findings: Formation of blisters in the epidermis and cells with ground-glass nuclei were observed in the blister cavities

**Fig. 4:**
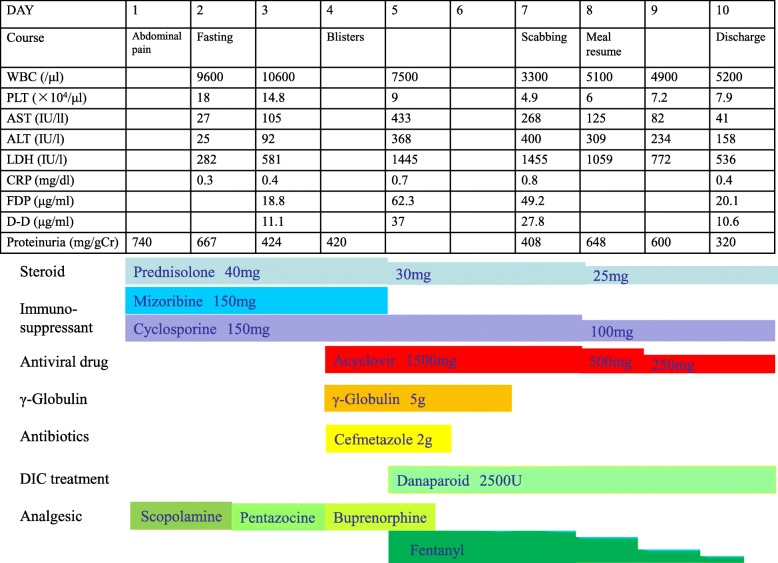
Clinical course: The top rows show the number of days from onset of abdominal symptoms to treatment and changes in other key data. The bottom rows show the treatment course of immunosuppressive therapy, acyclovir, gamma globulin, antibiotics, disseminated intravascular coagulation treatment, and analgesics. Abbreviations: WBC, white blood cell; PLT, platelet; AST, aspartic aminotransferase; ALT, alanine aminotransferase; LDH, lactate dehydrogenase; CRP, C-reactive protein; FDP, fibrinogen degradation products; D-D, D-dimer; DIC, disseminated intravascular coagulation

Clinical course: Despite pronounced abdominal pain, the vital signs were stable, there were no peritoneal irritation signs in the abdominal findings, and the tests and imaging lacked any serious findings. Cefmetazole (2 g/day) was administered as a prophylactic antibiotic, pain relief was provided with opioid analgesics, and the patient’s condition was carefully monitored as the cause of pain could not be identified at this point. On the morning of Day 80, a large number of small blisters appeared on the face and trunk, which are characteristic of varicella. Skin lesion biopsy revealed that blisters had formed within the epidermis, and cells with ground-glass nuclei were seen in the blister cavities (Fig. 3). Polymerase chain reaction results were positive for VZV. The blood VZV-DNA numbers had increased markedly. Initially, this patient had tested positive for VZV IgG, indicating that she had previously been infected with VZV, and the first VZV IgM test was negative on Day 80. On Day 86, paired sera samples were positive for VZV IgM. On the basis of these results (Table 2), the cause of the abdominal pain and skin lesions observed on Day 80 were confirmed to be VDVZV. The treatment course is shown in Fig. 4. Acyclovir (1500 mg/day) and immunoglobulin (5 g/day) were immediately administered intravenously, and the dosages of prednisolone and immunosuppressant were reduced. Liver function further worsened temporarily, disseminated intravascular coagulation was found and treatment was started. The abdominal pain quickly resolved, and the platelet count and liver function improved within 1 week of starting acyclovir. The patient was subsequently discharged. After discharge, on Day 104, we performed a repeat abdominal CT scan. This revealed mildly increased fat tissue density around the celiac artery and root of the superior mesenteric artery to reach normal levels (Fig. 2b). Retrospective evaluation of bed management revealed that another patient with herpes zoster had been in the same hospital room prior to the onset of VDVZV in this patient; therefore, it is highly likely that this was the source of the VZV infection.

**Table 2:** VZV tests

VZV antibody titer	Day 80	Day 86
VZV IgM/EIA	0.29 (−)	3.43 (+)
VZV IgG/EIA	7.5 (+)	23.0 (+)
Blood VZV DNA load	200,000 Copy	
Skin biopsy VZV PCR	+	

*EIA* enzyme immunoassay, *PCR* polymerase chain reaction, *VZV* varicella zoster virus

## Discussions and conclusions

We report the case of a patient who had previously been infected with VZV, but probably became re-infected with VZV during immunosuppressive therapy for membranous nephropathy, which led to the development of VDVZV. With careful diagnosis and management, we successfully treated the patient. It is generally considered that people with specific immunity to VZV will not become re-infected, although one study reported that four out of 62 people with VZV-specific antibodies (three of whom were immunologically healthy) who came into contact with a VZV-infected person subsequently developed varicella [10]. There are also data indicating that 4.15 out of 1000 people with specific immunity are re-infected with VZV per year [11]. In immunocompromised patients, the disease type at reoccurrence is not limited to herpes zoster; VZV can also present as varicella. Steroid administration generally increases the frequency and severity of VZV infection [12], and steroid administration of 61 days or longer is a risk factor. Women are reported to have a 25% higher incidence than men [11], but the reason for this sex-related difference is unknown. Furthermore, varicella is localized to the skin in immunologically healthy people, but the lesions are not similarly limited in immunocompromised patients, who can also develop VDVZV. A report has demonstrated that relapse was an onset factor for VDVZV in three out of four cases [3]. The patient in the present study developed VDVZV despite testing positive for VZV IgG, which suggests that this was re-infection due to the presence of a patient with herpes zoster in the same hospital room prior to the onset of the condition. Therefore, we conclude that VDVZV can occur in immunocompromised patients through VZV relapse, first-time infection, or re-infection.

The symptoms of the present case were characteristic: a sub-ileus profile with intense abdominal pain but no peritoneal irritation signs or lesions (such as perforations, occlusion, or thrombosis) and extremely mild elevation of leukocytes and inflammatory response, liver dysfunction, and disseminated intravascular coagulation (DIC) test findings. The abdominal pain preceded the skin rash.

Immunocompromised patients, particularly those with underlying blood diseases or kidney transplant recipients, are susceptible to VDVZV. Gastrointestinal symptoms, including abdominal pain, intestinal obstruction, vomiting, and diarrhea, precede the skin rash by a few to 10 days [13, 14], and the incidence of abdominal pain is high, occurring in 82–100% of cases [13, 15]. In a report on 15 VDVZV cases in Japan [15] (mean age was 27.9 years, 14 men and one woman), the underlying diseases were blood diseases (*n* = 12), kidney transplant (*n* = 2), and kidney disease (*n* = 1), and 13 of the patients were using steroids and/or immunosuppressants. The initial symptom was abdominal pain in 14 patients and back pain in one patient, and CT findings were almost completely normal. However, increased CT values of abdominal perivascular adipose tissue were observed in one case, erosion was evident by endoscopy in some cases, and some cases presented with a rash. The mean time from initial symptoms to appearance of the rash was 4.6 days. Narcotics were used for analgesia in five patients, and diagnostic methods included identification of the rash and anti-VZV IgM/IgG, serum VZV-DNA, serum VZV antigens, PCR from lesion site, and Tzanck tests. The mean time from initial symptoms to starting treatment was 4.5 days; all patients were treated with acyclovir and four patients were also treated with gamma globulin. The outcome was reported as death in four cases, and it was reported that the mean time from initial symptoms to starting treatment was 4.2 days for the 11 patients who survived, while a mean time of 5.5 days was reported for those who died. These results reiterate the importance of prompt treatment. In the present case, it was 3 days from the onset of abdominal pain to the appearance of the rash, diagnosis of VDVZV, and initiation of treatment. Until the rash appears, VDVZV is often diagnosed as abdominal pain of unknown etiology. It has been reported that the blood VZV-DNA concentration for herpes zoster is, on average, 1710 copies compared with an average of 214,214 copies for VDVZV. This disparity could be used as a method for diagnosis during the time before the appearance of the rash [16], and measuring blood VZV-DNA numbers when VDVZV is suspected could be useful. The imaging findings are usually non-specific; increased lipid concentration around the superior mesenteric artery by CT has been reported [17], and the present case had similar CT findings (Fig. 1).

Since VZV is known to be associated with vasculitis and giant cell arteritis, it is possible that the clinical findings relate to the arteritis accompanying VZV infection [18, 19]. In the context of autoimmunity or immunodeficiency, VZV vasculitis often occurs in the cerebral blood vessels; [20] however, it can occur in the abdominal blood vessel, as was observed in the present case.

There have been reports of upper gastrointestinal bleeding or herpes-like lesions on the mucosal or serosal side of the gastrointestinal tract [21, 22]. Pain management is difficult in such cases and may require opioid analgesics, but there are also reports where even large doses of opioids were not sufficient [14, 17]. There is a theory that the abdominal pain is caused by proliferation of the VZV on the celiac and mesenteric ganglia [23], but there are number of different theories [13, 21] and the details are still unknown. This study had some limitations. Specifically, we did not perform gastroscopy and intestinal endoscopy, and no viral genotyping was available to allow differentiation of disseminated zoster (reactivation) from re-infection.

The prognosis of VDVZV is poor; often becoming much more serious with complications of DIC, encephalitis, pneumonia, and intestinal necrosis; and the mortality rate is reported to be extremely high, at approximately 55% [24]. Recently, the incidence of VDVZV in Japanese patients receiving allogenic hematopoietic stem cell transplantation was reported to be 0.8%, and the mortality rate was 20% [4]. Delayed diagnosis has fatal outcomes, sometimes within a few days [2, 23]. Therefore, awareness of this condition as a severe infection is vital.

Infection prevention is important and measures must be taken to ensure that immunocompromised patients do not come into contact with VZV. In the USA, a live attenuated vaccine is recommended to prevent herpes zoster and postherpetic neuralgia in general patients aged 60 years and older, and there is also a recombinant vaccine recommended for individuals 50 years and older [25–27]. Administration of live vaccines is normally contraindicated as a prophylactic measure for immunocompromised patients, but their use after organ transplant has been reported [28]. However, pediatricians do not consider the administration of live vaccines to be absolutely contraindicated for transplant recipients, and there are reports that such patients may develop a fever and rash, but do not subsequently develop VZV infection even after exposure to the virus [29, 30]. Therefore, there is no consensus of opinion.

Prophylactic oral administration of acyclovir has been reported to inhibit VZV activation in hematopoietic stem cell transplantation cases [31, 32], but the inhibitory effect has been demonstrated to cease once administration is stopped [33]. Acyclovir is excreted via the kidneys; therefore, ongoing prophylactic administration requires caution in patients with renal insufficiency. The KDIGO guidelines recommend intravenous injection of VZV immunoglobulin or immunoglobulin within 96 h as a preventative measure to counteract exposure of kidney transplant recipients to VZV [34]. The same guidelines advocate a combination of intravenous administration of acyclovir and temporary reduction of immunosuppressants as a treatment for VZV [34]. A report of the VZV-related death of a patient during immunosuppressive therapy for kidney disease treatment has been published; however, the acyclovir dose was too low (250 mg/day) and the steroid dose was not reduced [2]. Awareness of VDVZV must be reiterated not only for patients with blood diseases and kidney transplant recipients, but also for patients with kidney disease undergoing immunosuppressive therapy. It is important to start treatment with a sufficient dose of acyclovir as soon as possible and to reduce the dose of immunosuppressive therapy.

## Data Availability

Not applicable.

## Abbreviation

VDVZV: Visceral disseminated varicella zoster virus
